# Age-period-cohort decomposition and projections of the frailty index among older adults in China

**DOI:** 10.3389/fpubh.2026.1806784

**Published:** 2026-04-15

**Authors:** Shuyin Zhang, Handong Li

**Affiliations:** School of Systems Science, Beijing Normal University, Beijing, China

**Keywords:** APC, China’s older population, CLHLS, frailty index, projections

## Abstract

**Introduction:**

The frailty index is a composite indicator of older adults’ health status. This study examines temporal trends in frailty among China’s older population and seeks to disentangle the respective roles of aging, cohort replacement, and period-specific environments.

**Methods:**

Using longitudinal data from the Chinese Longitudinal Healthy Longevity Survey (CLHLS) and drawing on the accelerated longitudinal design framework, we decompose changes in frailty along the age–period–cohort (APC) dimensions. We estimate the marginal contributions of age, survey period, and birth cohort to frailty patterns among older Chinese adults and, on this basis, generate projections of future frailty trends.

**Results:**

First, the frailty index increases strictly monotonically with age. The cohort effect exhibits an overall declining trend, such that later-born cohorts show lower frailty at the same age and in the same period. The period effect shifts upward over the sample window, suggesting that the influence of macro-level environments and institutional change on frailty is not a simple unidirectional health gain. Second, frailty displays pronounced gender and urban–rural disparities: rural men have the lowest overall levels, whereas urban women rise more rapidly at advanced ages and remain the most frail. Moreover, the urban–rural gap among women widens faster than that among men, and within-city gender disparities also intensify more rapidly than within rural areas; advanced old age emerges as a critical interval in which urban–rural and gender inequalities interact and amplify. Third, frailty among China’s future older population is projected to worsen continuously, albeit at different rates across groups. The largest increase is expected among urban older women, followed by rural older women, while frailty among rural older men is projected to surpass that of urban older men after 2035. Across years, the upward shift is generally larger for rural groups than for urban groups and is more pronounced at advanced ages, implying that the future burden of frailty may be increasingly concentrated among rural older adults.

**Discussion:**

These findings highlight the importance of an APC-informed and ALD-based perspective for interpreting frailty dynamics and for producing structurally grounded projections. The projected widening of rural disadvantage at advanced ages underscores the need for forward-looking public health and long-term care planning with attention to urban–rural and gender inequalities.

## 1 Introduction

In recent years, population ageing in China has continued to deepen, with the size of the older population expanding rapidly alongside structural changes such as persistently low fertility, the shrinking of household size, and intensified population mobility (1). In the context of a deeply ageing society, older adults’ health problems are often no longer characterized by the onset and treatment of a single disease; rather, they increasingly manifest as a long-term process involving multimorbidity, functional decline, cognitive deterioration, and the cumulative accumulation of disability risk (2). Traditional models of health governance therefore face new challenges, implying that the core of public health demand is shifting from controlling single diseases to managing functional deterioration and care-related risks. Against this backdrop, frailty—an integrative health state reflecting multisystem reserve depletion and reduced resilience to stressors—has been widely used to characterize the continuous transition of older individuals from relatively healthy conditions to high-risk disability (3, 4). Compared with single chronic-disease indicators or conventional demographic measures of age structure, frailty better captures the multidimensional vulnerability of health in later life and is robustly associated with mortality, disability, hospitalization, long-term care needs, and related outcomes (5–9). Therefore, examining frailty from a demographic perspective not only enables a more fine-grained understanding of changes in health status among older populations, but also provides more direct evidence for public health resource allocation, the provision of integrated medical–care services, and the development of long-term care systems.

However, changes in frailty levels among older adults reflect not only the physiological ageing process over the life course, but are also strongly shaped by long-term factors that vary across birth cohorts, including early-life nutrition, educational attainment, labor intensity, and exposure to disease. Meanwhile, within the survey period, improvements in the health-care system, the expansion of social security, and shocks from public health events may also substantially alter frailty levels among individuals of the same age. If analysis relies solely on cross-sectional age profiles or simple time trends, cohort replacement or period shocks can easily be mistaken for pure age-related change. This may bias interpretations of the mechanisms underlying frailty dynamics and undermine the validity of forecasting and policy evaluation. This issue is particularly salient in the Chinese context. China’s older population has experienced highly heterogeneous historical and developmental stages, and different birth cohorts exhibit marked disparities in early-life conditions, nutritional status, educational access, occupational trajectories, and health-care availability (10). At the same time, the rapid expansion of the health-care system and improvements in primary public health capacity over the past two decades have also reshaped the overall distribution of frailty at the period level (11). Therefore, to address key population-level questions—such as whether frailty among older adults has become more severe or has improved, and how urban–rural and gender disparities evolve across age groups—it is necessary to situate frailty dynamics within an age-period-cohort (APC) framework and conduct a structural decomposition that accurately identifies the respective contributions of these three effects to frailty among older adults.

Building on the above context, this paper focuses on the dynamics of frailty among China’s older population. Using longitudinal survey data, we decompose changes in frailty within an APC framework and estimate the marginal contributions of age, survey period, and birth cohort to frailty levels. On this basis, we further project frailty trends by incorporating future population age-structure changes. Meanwhile, given the persistence of China’s urban–rural dual structure and long-standing gender disparities, we compare frailty trajectories across urban–rural and gender subpopulations and examine how these differences vary with age. The goal is to provide quantitative evidence with stronger structural interpretability and forward-looking value to inform assessments of public health needs and policy design in the context of a deeply ageing society.

## 2 Literature review

### 2.1 Conceptualization and measurement of frailty

In geriatric medicine, frailty cannot be explained by any single disease (12), yet it is closely associated with adverse health outcomes and substantial increases in health-care expenditures (4). Frailty is widely regarded as a syndrome characterized by declines across multiple physiological systems in later life and has distinctive value in both clinical and public health contexts (13–17). Consensus statements and working-group documents oriented toward clinical practice define frailty as a clinical state in which exposure to a stressor markedly increases the risk of dependency or death, and they have promoted the incorporation of frailty assessment into routine screening and stratified management pathways (3, 18).

Two classic approaches to measuring frailty are commonly used. The first is the frailty phenotype proposed by Fried et al. (19–21). The Fried phenotype emphasizes clinical characteristics such as weight loss, exhaustion, reduced grip strength, slowed gait speed, and low physical activity (19, 22, 23). Its main advantage is parsimony, enabling relatively rapid assessment of vulnerability among older adults (24). The second approach is represented by the frailty index (FI) (25, 26). Grounded in the deficit-accumulation framework, the FI aggregates multidimensional health deficits—including diseases, functional impairments, psychological problems, and cognitive deficits—into a continuous indicator ranging from 0 to 1 (27–29). The FI was originally developed by Mitnitski, Mogilner, and Rockwood based on the Canadian Study of Health and Aging. It considered 92 candidate health deficits spanning symptoms, signs, abnormal laboratory findings, chronic conditions, and disability, and summarized them into a single measure (25). Subsequent studies have further demonstrated the FI’s robust statistical properties and its value for risk stratification across diverse data settings (30). Rockwood et al. showed that reducing the number of candidate deficits to 30 does not compromise the predictive validity of the FI (31). Other research suggests that candidate deficits need not be restricted to particular types of symptoms, signs, disabilities, or laboratory abnormalities; rather, they should satisfy general principles, including being age-related, health-relevant, covering major physiological systems, and not saturating too early (32, 33). The FI has been widely used in recent years to examine associations between frailty and outcomes such as mortality, disability, and health-care utilization, and it has increasingly been incorporated into research frameworks assessing population health, healthy life expectancy, and long-term care needs among older adults (34–36).

Beyond these two major approaches, a range of scales and screening tools has been developed for different application settings. For example, the Clinical Frailty Scale (CFS) facilitates rapid stratification and clinical communication (27); comprehensive instruments emphasize multidimensional assessment and psychometric evidence (37, 38); and community- and primary-care screening tools—such as PRISMA-7 (39), SHARE-FI (9) and FRAIL—highlight low cost, few items, and scalability (40), while the Groningen Frailty Indicator (GFI) reflects early European approaches to community-based screening (41). Furthermore, Jones et al. proposed a pathway that translates comprehensive geriatric assessment (CGA) directly into a deficit-accumulation measure, strengthening the linkage between FI construction in clinical practice and its implementation in survey data (42).

### 2.2 Research on frailty among older adults in China

Chinese scholars have conducted extensive research on frailty among older adults based on the frailty index (FI), with primary emphases on (i) the adverse consequences of frailty, (ii) determinants of frailty, and (iii) structural disparities.

First, evidence on adverse outcomes. Gu et al. (43) constructed a multidimensional deficit-based FI using CLHLS data and reported a significant association between frailty and mortality risk among the oldest-old, with the frailty–mortality relationship remaining robust after adjustment for multiple covariates. Shi et al. (44), using the Beijing Longitudinal Study of Aging (BLSA), showed that deficit accumulation increases with age and can predict mortality. Fang et al. (45) found that falls and fractures are common among older adults and are associated with frailty; moreover, frailty remained independently associated with mortality after controlling for confounding. Using a large prospective cohort from the China Kadoorie Biobank, Fan et al.^[17]^further demonstrated that FI is associated with both all-cause and cause-specific mortality, and that these associations are independent of chronological age. Based on CHARLS data, Xu et al. (46) showed that frailty substantially reduces the likelihood of recovery after disability, serving as an important factor distinguishing trajectories from disability to recovery versus persistent disability. Fan et al. (47) reported that higher frailty levels are linked to elevated risks of outpatient visits and hospitalization, and that frailty is significantly associated with catastrophic health expenditure. In addition, Cai et al. (48) found that prefrailty and frailty are associated with adverse outcomes such as falls, severe falls, more frequent health-care use, and readmission. Chen et al. (49) reported a higher risk of falls among individuals with cognitive frailty and developed a fall-risk prediction model accordingly.

Second, determinants of frailty. The literature generally agrees that frailty increases with age (50) and is associated with socioeconomic status (51), chronic disease burden (52), lifestyle factors (53), and social support (54, 55). Beyond these factors, cognition and diet have also received attention. For example, Zhao et al. (56) examined the longitudinal relationship between frailty and cognitive impairment using CLHLS data and suggested that the two may be mutually intertwined, jointly shaping subsequent health risks. Using CLHLS, Wang et al. (57) found that greater dietary diversity is associated with lower frailty risk, highlighting the public health relevance of diet structure as a modifiable behavioral factor. Using CHARLS, Xu et al. (58) reported that sarcopenia is significantly and independently associated with frailty and prefrailty.

Third, structural disparities in frailty. Yu et al. (59) compared urban and rural samples in CLHLS and found higher FI levels and a more pronounced survival disadvantage among rural older adults; they linked these patterns to urban–rural disparities in socioeconomic conditions and health-care resources. Using CHARLS, Wu et al. (60) estimated the prevalence of frailty and prefrailty among adults aged 60 and above with the physical frailty phenotype and documented stratified differences by region, urban–rural residence, and educational attainment. Their findings indicate that frailty is not merely a product of biological ageing but is closely connected to structural inequality. Consistent with this view, Zhou et al. (61) conducted a systematic review and meta-analysis of frailty prevalence among community-dwelling older adults in China, providing pooled estimates of overall frailty prevalence and prefrailty proportion, and reporting that factors such as age, gender, marital status/living alone, education, and region/rural residence are associated with higher frailty risk.

Finally, other research themes. Using CLHLS, Liu et al. (62) characterized transitions across frailty states and showed that frailty is a dynamic process, with different transition patterns associated with mortality. Guo et al. (63) identified distinct frailty trajectory classes in a national cohort of community-dwelling older adults and examined how demographic, socioeconomic, and behavioral factors relate to trajectory membership. Using CLHLS, Bi et al. (64) employed a dual-trajectory model to jointly model the co-evolution of social participation and FI, identifying coupled trajectory patterns and shared determinants; they argued that social participation may not only be correlated with frailty but may also evolve synchronously with the frailty process. Further, Liu et al. (65) incorporated changes in FI directly into mortality-risk analyses and found that short-term (e.g., three-year) increases in FI are significantly associated with subsequent mortality risk, with evidence of nonlinearity.

Despite these contributions, the existing literature has two notable limitations. First, most studies focus primarily on age-related influences, while research on period and cohort factors shaping population-level frailty remains limited. Second, there is a lack of work examining trends in frailty among China’s older population explicitly within an age-period-cohort framework. Addressing this gap is important for allocating public health resources and strengthening the pension and long-term care system under conditions of deep population ageing.

## 3 Data and methods

### 3.1 Data source

This study uses multiple waves of the Chinese Longitudinal Healthy Longevity Survey (CLHLS) (2000, 2002, 2005, 2008, 2011, 2014, and 2018). Each wave includes both newly recruited respondents and re-interviewed individuals, so the dataset exhibits a hybrid structure that combines replenished cross-sectional samples with longitudinal follow-up. We harmonize all waves into a long-format panel, where each observation corresponds to an individual’s interview record in a given wave. Here, FI denotes the frailty index, age denotes age at interview, and cohort denotes birth cohort, which remains invariant for the same respondent across waves; period represents period information (survey year).

The CLHLS employs a multiwave follow-up design with continuous sample replenishment, resulting in a mixed data structure that simultaneously contains longitudinal tracking information and repeated cross-sectional information. In studies of older adults’health, data structure determines which temporal mechanisms can be identified. Cross-sectional data can describe age differences at a given time point but cannot disentangle age effects from cohort differences (66, 67). Conventional longitudinal data can capture within-individual change over time; however, constrained by study duration, they typically cover only a limited age window and thus cannot directly recover continuous trajectories across a broad age range (68, 69). Against this backdrop, the accelerated longitudinal design (ALD) has become a key approach for combining the strengths of these two data types. Its core idea is to follow multiple birth cohorts that enter observation at different starting ages and to integrate these short longitudinal windows within a statistical model, thereby reconstructing developmental trajectories over a much longer age span within a limited follow-up period (70–73). Accordingly, in research on continuous health outcomes such as frailty, ALD not only avoids inferring life-course change solely from cross-sectional differences but also mitigates the time and sample-cost constraints inherent in long-term follow-up of a single cohort (70, 74).

### 3.2 Construction of the frailty index

This study adopts a deficit-accumulation framework to construct the frailty index (FI). For individual i in wave t , the k-th deficit indicator is denoted by $d_{\mathrm{itk}}$ and takes value $d_{\mathrm{itk}}\in \{0,1,\mathrm{NA}\}$ , where 1 indicates the presence of a deficit, 0 indicates no deficit, and NA indicates missing information for that item. Because the CLHLS questionnaires are not fully identical across waves, we restrict candidate deficits to items that are available in all survey waves and use them as sources for deficit construction.

Following Yang and Gu (75), after screening, we include deficits covering cognitive function, psychological wellbeing, self-rated health, interviewer-rated health, whether the respondent experienced a serious illness in the past 2 years, visual and hearing impairment, six activities of daily living (ADL) items, eight instrumental activities of daily living (IADL) items, 11 chronic disease items, and five functional limitation items (four functional limitation items in the 2000 wave), yielding 37 indicators in total (36 in 2000), as summarized in Table 1. To ensure comparability across survey waves in the presence of minor questionnaire differences, we restrict candidate deficits to items that can be harmonized to a consistent binary coding scheme across all waves, and we apply wave-consistent recoding rules so that each deficit indicator has the same substantive meaning over time.

**Table 1:** Deficit items and specific deficits.

Deficit domain	Specific deficit
Basic cognition	cognitive function
Psychological status	psychological wellbeing
Health status	self-rated health
	interviewer-rated health
Serious illness status	serious illness in the past 2 years
Vision and hearing status	visual impairment
	hearing impairment
ADL	Ability to bathe independently
	Ability to dress independently
	Ability to use the toilet independently
	Ability to move indoors independently
	Ability to control urination and defecation
	Ability to eat independently
IADL	Ability to visit neighbors independently
	Ability to go shopping independently
	Ability to cook independently
	Ability to wash clothes independently
	Ability to walk 2 li continuously
	Ability to lift a 5-kg weight
	Ability to squat down and stand up three times continuously
	Ability to use public transportation independently
Chronic disease items	Hypertension
	Diabetes
	Heart disease
	Stroke and cerebrovascular disease
	Bronchitis, asthma, or pneumonia
	Cataract
	Glaucoma
	Cancer
	Gastric or duodenal ulcer
	Parkinson’s disease
	Arthritis
Functional limitation items	Touching the back of the neck with the hand
	Touching the lower back with the hand
	Raising the arm upward
	Ability to stand up from a chair independently
	Ability to pick up a book from the floor

Missingness is handled in two steps. First, at the individual–wave level, we compute the proportion of missing deficit items among the full candidate set and exclude observations with excessive missingness (i.e., when more than 20% of deficit items are missing for an individual in a given interview), to avoid unstable FI values driven by insufficient information. Second, for the remaining observations, we adopt a partially strict dynamic-denominator approach: if a specific deficit item is missing for an individual in that interview, the item is excluded from both the numerator and the denominator, such that the FI is computed from the set of valid (non-missing) deficits observed in that interview. This approach preserves sample size while preventing missing items from artificially inflating or deflating the FI and maintains measurement consistency across waves under the harmonized deficit set.

Let $D_{\mathrm{it}}=\sum_{k=1}^{K}1(d_{\mathrm{itk}}=1)$ denote the number of observed deficits for individual i in wave t, and $N_{\mathrm{it}}=\sum_{k=1}^{K}1(d_{\mathrm{itk}}=\{0,1\})$ denote the total number of valid (non-missing) deficit items observed. The frailty index is then defined as $FI_{\mathrm{it}}=\frac{D_{\mathrm{it}}}{N_{\mathrm{it}}},FI_{\mathrm{it}}\in [0,1]$ .

### 3.3 APC decomposition method

As a foundational methodological approach in demographic research, age–period–cohort (APC) analysis is used to distinguish the respective contributions of individual life-course processes (age effects), macro-historical contexts (period effects), and generational social imprints (cohort effects) to long-term changes in an outcome variable (76, 77). Frailty, as an integrated representation of multidimensional health deficits, exhibits a pronounced age-related increase and is also highly sensitive to intercohort differences in life histories and to changes in institutional environments. Incorporating frailty into an APC framework therefore enhances the structural interpretability of long-term trends and provides a more defensible basis for projection, because future frailty levels can be represented as the superposition of an age trajectory and extrapolated cohort and period components rather than as a mechanical extension of aggregate trends. A central challenge of APC analysis, however, is the exact linear dependency among age, period, and cohort, which renders the decomposition non-identifiable under a purely linear and fully parametric specification. To address this challenge in a transparent and reproducible way, the present study adopts a semi-parametric APC formulation embedded in a generalized additive mixed model (GAMM): the age and cohort components are represented by penalized regression spline smooths, while the period component is represented by survey-year fixed effects. Under the mgcv implementation, smooth components are subject to an explicit centering (sum-to-zero) constraint in the sample, which removes the indeterminacy arising from arbitrary shifts between the intercept and smooth functions and thereby prevents non-uniqueness due to additive confounding. Estimation proceeds via penalized likelihood, where smoothness penalties regularize the functional components and the random-intercept structure accounts for within-individual correlation; smoothing parameters and variance components are selected by restricted maximum likelihood (REML), yielding a stable decomposition under the stated constraint-and-penalty structure. Accordingly, the extracted age, cohort, and period components should be interpreted as a model-based APC decomposition identified by explicit centering and regularization, rather than as unconstrained pure linear APC coefficients. This strategy is conceptually related to, but distinct from, other APC identification approaches that impose different identifying assumptions, such as the intrinsic estimator (IE) (78) and related constraint-based solutions, hierarchical APC (HAPC) (79) formulations based on cross-classified random effects, and APC specifications implemented in generalized additive modeling frameworks (APC-GAM) (80). These approaches differ primarily in where identification is imposed, namely through algebraic estimability constraints, hierarchical variance-structure assumptions, or smoothness assumptions on the temporal dimensions. In contrast, the present framework makes its identifying assumptions explicit as (i) centering constraints on smooth components and (ii) penalized estimation with REML-selected smoothness, while retaining period as survey-year fixed effects because the number of waves is limited and the analysis requires wave-linked period shifts that can be directly extrapolated in the forecasting stage.

To simultaneously model (i) the nonlinear age pattern of FI, (ii) systematic differences across birth cohorts, and (iii) period differences across survey waves, while accounting for within-individual correlation induced by repeated measurements, we fit a generalized additive mixed model (GAMM) within each subgroup (Equation 1).


$$FI_{\mathrm{it}}=\beta_{0}+f(a_{\mathrm{it}})+h(c_{i})+\gamma_{t}+b_{i}+\in_{\mathrm{it}},i=1,2,\ldots ,N_{g}$$ (1)

Here, $\beta_{0}$ is the intercept; f(⋅) is a smooth function for the age effect, capturing the nonlinear trajectory of the frailty index (FI) over age—i.e., the average difference in FI attributable to age variation, conditional on the same birth cohort and the same survey wave; h(⋅) is a smooth function for the cohort effect, controlling for systematic differences across birth cohorts—i.e., the average difference in FI across birth years, conditional on the same age and the same survey wave; $\gamma_{t}$ denotes the period effect, controlling for overall level differences across survey years—i.e., the average shift in FI associated with the broader environment of different survey years, conditional on the same age and birth year; b is an individual-specific random intercept that captures stable between-person heterogeneity and accounts for within-person correlation due to repeated measurements; and $\in_{\mathrm{it}}$ is the residual term. The random component is assumed to follow: $b_{i}\sim N(0,\sigma_{b}^{2})$ , $\in_{\mathrm{it}}\sim N(0,\sigma^{2})$ .

Within the mgcv framework in R, the smooth terms are represented by expanding them over spline basis functions, as shown in Equation 2.


$$f(a)=\sum_{j=1}^{K_{a}}\alpha_{j}B_{j}(a),h(c)=\sum_{m=1}^{K_{c}}\delta_{m}Q_{m}(c)$$ (2)

Here, $B_{j}(\cdot )$ and $Q_{m}(\cdot )$ denote spline basis functions; $\alpha_{j}$ and $\delta_{m}$ are the coefficients to be estimated; and $K_{a}$ and $K_{c}$ are the pre-specified upper limits of the basis dimensions for the corresponding smooth terms. In addition, to ensure model identifiability and to avoid non-uniqueness arising from the intercept being confounded with the overall level of the smooth terms, mgcv imposes a centering constraint on each smooth term such that it has zero mean in the sample sense Equation 3.


$$\sum_{\mathrm{it}}f(a_{\mathrm{it}})=0,\sum_{i}h(c_{i})=0$$ (3)

Accordingly, the intercept $\beta_{0}$ represents the baseline level under the reference survey wave after the smooth terms have been centered at zero; the period coefficient(s) $\gamma_{t}$ capture the mean shift relative to the reference wave.

Moreover, under the Gaussian (identity-link) specification, estimating the above model is equivalent to minimizing a penalized objective function, as shown in Equation 4.


$$\begin{array}{l} \min_{\beta_{0},\sigma ,\alpha ,\delta ,b}\frac{1}{\sigma^{2}}\sum_{\mathrm{it}}{\left(FI_{\mathrm{it}}-\beta_{0}-f(a_{\mathrm{it}})-h(c_{i})-\gamma_{t}-b_{i}\right)}^{2}\\ +\lambda_{a}\alpha^{Τ}S_{a}\alpha +\lambda_{c}\delta^{Τ}S_{c}\delta +\frac{1}{{\sigma_{b}}^{2}}\sum b_{i}^{2}\; \end{array}$$ (4)

Here, the second and third terms are smoothness penalties that control the roughness of the curves, with $\lambda_{a}$ and $\lambda_{c}$ denoting the smoothing parameters; the fourth term is a regularization penalty for the random intercept. The smoothing parameters and variance components are estimated automatically under the REML framework, thereby balancing goodness of fit and the smoothness of the estimated functions. To assess the robustness of the GAMM results to smoothing specifications, we conducted a sensitivity analysis by varying the spline basis dimension and the penalty inflation factor within a plausible range while keeping the model structure and sample unchanged. For each specification, we re-estimated the model and compared the resulting age, cohort, and period components with the baseline estimates. The results show that all three effect curves are highly stable across specifications. The curve-wise correlations with the baseline are consistently close to one in all subgroups, and the maximum pointwise absolute deviations remain small. This indicates that the main findings are not driven by a specific smoothing setting, but reflect robust patterns supported by the data.

After model fitting, we obtain three components: the age-effect curve, the cohort-effect curve, and the period effect. Under an accelerated longitudinal design, the sample consists of repeatedly observed individuals together with replenishment samples added at each wave. As a result, the distributions of birth cohorts and survey years are uneven in the data. If we present $\hat{f}(a)$ under a fixed combination (c,t) , the resulting curve is a conditional age trajectory specific to a given cohort and period context, and thus cannot represent the population-level mean pattern. To obtain an average age–frailty curve with demographic relevance, it is necessary to marginalize over (c,t) .

Let the subgroup be indexed by g. For any age a within subgroup g, the marginalized age curve is defined as an observation-weighted average over the empirical joint distribution of (c,t) (as in Equation 5).


$$\mu_{g}(a)=\sum_{(c,t)\in \delta_{g}}w_{g}(c,t)[{\hat{\beta }}_{0,g}+{\hat{f}}_{g}(a)+{\hat{h}}_{g}(c)+{\hat{\gamma }}_{t,g}]$$ (5)

As shown in (Equation 6), $\delta_{g}$ denotes the set of observed (c,t) combinations in subgroup g ; $w_{g}(c,t)$ is the weight assigned to each combination, and $n_{g}(c,t)$ is the number of observations in subgroup g that fall into (c,t) .


$$w_{g}(c,t)=\frac{n_{g}(c,t)}{\sum_{(c,t)\in \delta_{g}}n_{g}(c,t)}$$ (6)

At each age a , $\mu_{g}(a)$ is a weighted average of multiple model-based predictions. In our implementation, we use the model-predicted standard errors and aggregate them during marginalization to construct confidence intervals for the age curve. This marginalized curve represents the average age trajectory of the FI after accounting for cohort and period differences.

### 3.4 Forecasting model for the frailty index

After estimating the age, cohort, and period effects, we further extend these three components to future years to generate projections of frailty levels among China’s older population. The cohort effect $\hat{h}(c)$ is identified over the range of birth cohorts observed in the sample. If future cohorts exceed the maximum observed birth year $c_{\max}$ that can be identified from the data, we extrapolate the cohort effect using a tail-linear extension, as specified in Equation 7:


$$\hat{h}(c)\approx \eta_{0,g}+\eta_{1,g}c$$ (7)

Based on this definition, the cohort effect is extrapolated accordingly (Equation 8).


$${\tilde{h}}_{g}(c)=\{\begin{array}{c} \hat{h}(c),c\leq c_{\max}\\ \eta_{0,g}+\eta_{1,g}c,c>c_{\max} \end{array}$$ (8)

For projecting the period effect { ${\hat{\gamma }}_{t,g}$ }, we proceed as follows. First, we take the discrete period-effect estimates $\left\{t,{\hat{\gamma }}_{t,g}\right\}$ obtained from model fitting as inputs and fit a smooth function $\;s_{g}(t)$ over calendar year. We then extend this smooth function to future years using Equation 9, yielding projected period effects ${\hat{\gamma }}_{t,g}$ .


$${\hat{\gamma }}_{t,g}\approx s_{g}(t),t\in {ℑ}_{g};{\hat{\gamma }}_{y,g}={\hat{s}}_{g}(y)$$ (9)

For forecasting, we specify a reference period $t^{*}$ and a reference cohort $c^{*}$ . Under the reference scenario $\left(t^{*},c^{*}\right)$ , we first define the age-specific baseline as (Equation 8). We then add the increments of future cohort effects and future period effects relative to the reference point to the age baseline (Equation 10).


$$FI_{g}^{\mathrm{ref}}(a)=\hat{E}(\mathrm{FI}|a,c=c^{*},t=t^{*})$$ (10)

At the population level, we further introduce projected age-specific population counts pop as weights and aggregate age-specific frailty predictions into a population-weighted mean frailty level for each subpopulation in each year (Equation 11).


$${\hat{\mathrm{FI}}}_{g}(a,y)=FI_{g}^{\mathrm{ref}}(a)+[{\tilde{h}}_{g}(y-a)-{\hat{h}}_{g}(c^{*})]+[{\tilde{\gamma }}_{y,g}-{\hat{\gamma }}_{t^{*},g}]$$ (11)

All analyses and model implementations are conducted in the R environment.

## 4 Empirical results

### 4.1 Data description and the distribution of the frailty index

**Figure 1:**
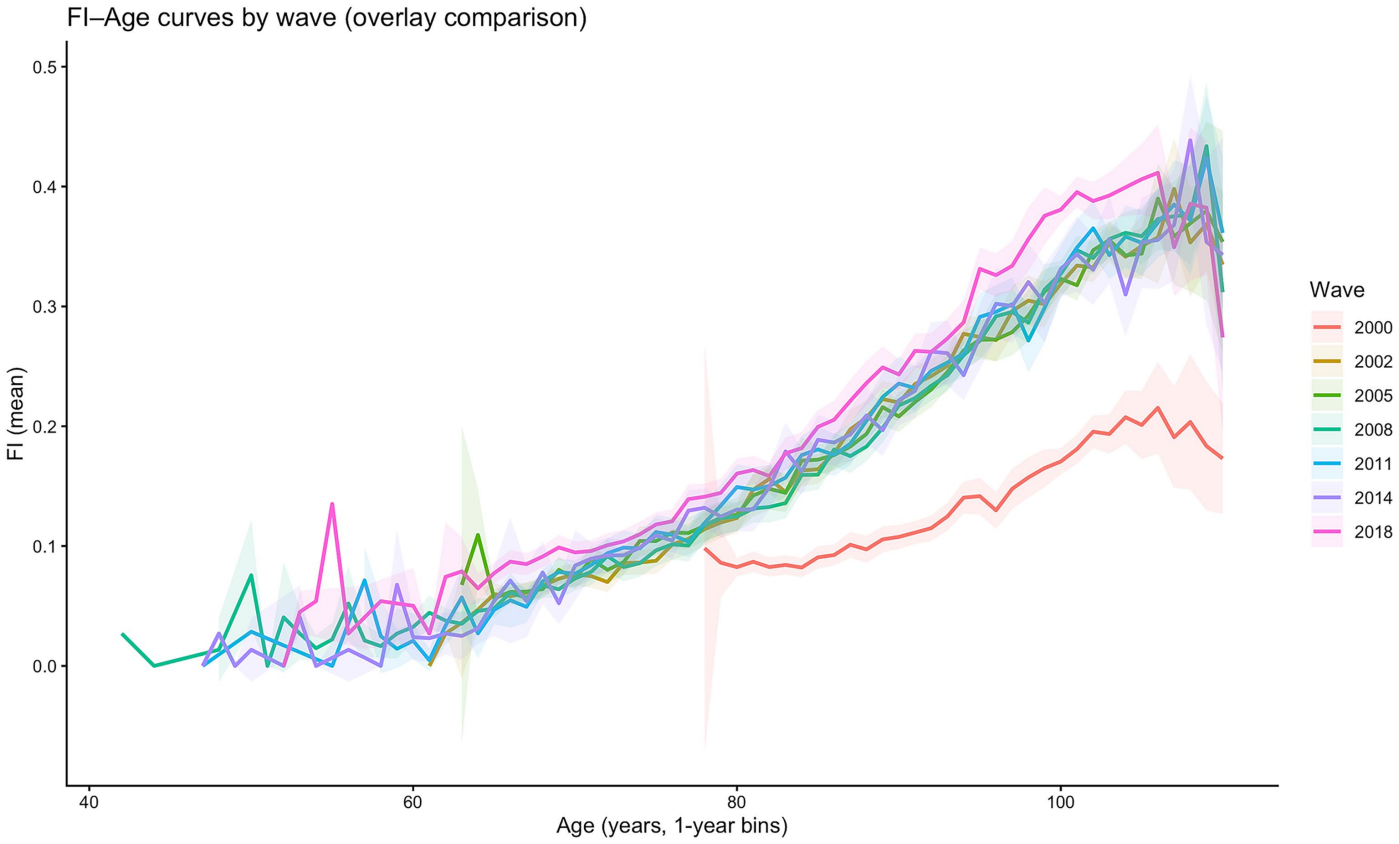
Distribution of the frailty index across survey waves.

Using the eight waves of CLHLS panel data, we compute the mean frailty index of older adults in China by age for each survey year, as shown in Figure 1. Figure 1 indicates that the frailty index in 2000 is markedly lower than in other years, whereas the distributions in the remaining waves appear more plausible. We interpret this 2000 deviation primarily as a wave-specific comparability issue rather than a substantive improvement in health. In particular, the 2000 questionnaire and coding regime differ from subsequent waves in several deficit items, and the operational thresholds for identifying deficits are relatively less stringent, which can mechanically shift the FI–age profile downward even after conditioning on age.

**Figure 2:**
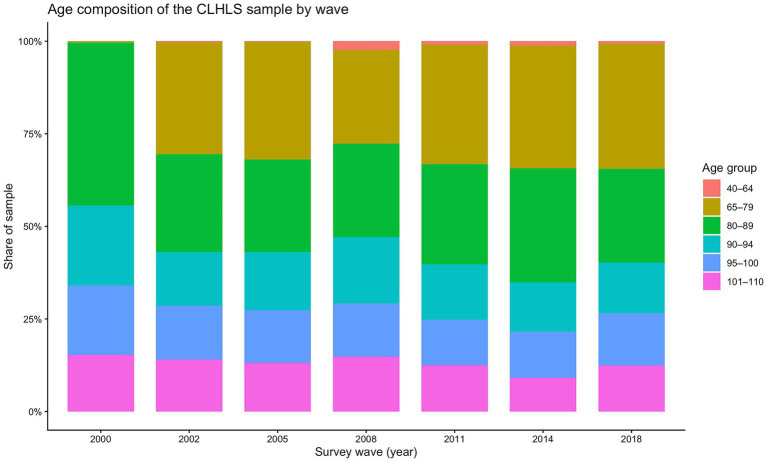
Distribution of the population age structure.

At the same time, the 2000 wave also exhibits a markedly different sample age structure. As documented in Figure 2, respondents in 2000 are disproportionately concentrated in the oldest-old ages, with much thinner coverage at the younger-old ages relative to later waves. This compositional discrepancy implies weaker overlap of age support between the 2000 wave and subsequent surveys. Importantly, however, because Figure 1 is based on age-specific means, the unusually low FI in 2000 cannot be explained by the wave’s older sample composition alone; instead, it more plausibly reflects measurement non-equivalence across waves. To avoid contaminating the APC decomposition with a one-off measurement regime shift and to preserve temporal comparability of FI, we exclude the 2000 wave from model estimation and retain only the survey waves of 2002, 2005, 2008, 2011, 2014, and 2018. In the sample, the numbers of observations at the younger-old and the extreme-old ages are relatively small; because sample size directly affects model fit, we restrict the age range for model estimation to 65–105 years.

Our analysis shows that, over the study period, the CLHLS panel contains a large proportion of respondents who were interviewed only once or who appear in only a small number of adjacent waves. Consequently, the share of a strictly complete panel is relatively low: longitudinal information is contributed primarily by a limited subset of repeatedly measured individuals, whereas cross-sectional representativeness relies mainly on the ongoing replenishment and sample-refresh mechanisms implemented at each wave. Table 2 reports the distribution of follow-up frequencies across the six waves in the analytical sample.

**Table 2:** Distribution of follow-up counts.

Number of follow-up waves	1	2	3	4	5	6
Percentage	64.1%	17.6%	8.7%	5.0%	3.0%	1.6%

**Figure 3:**
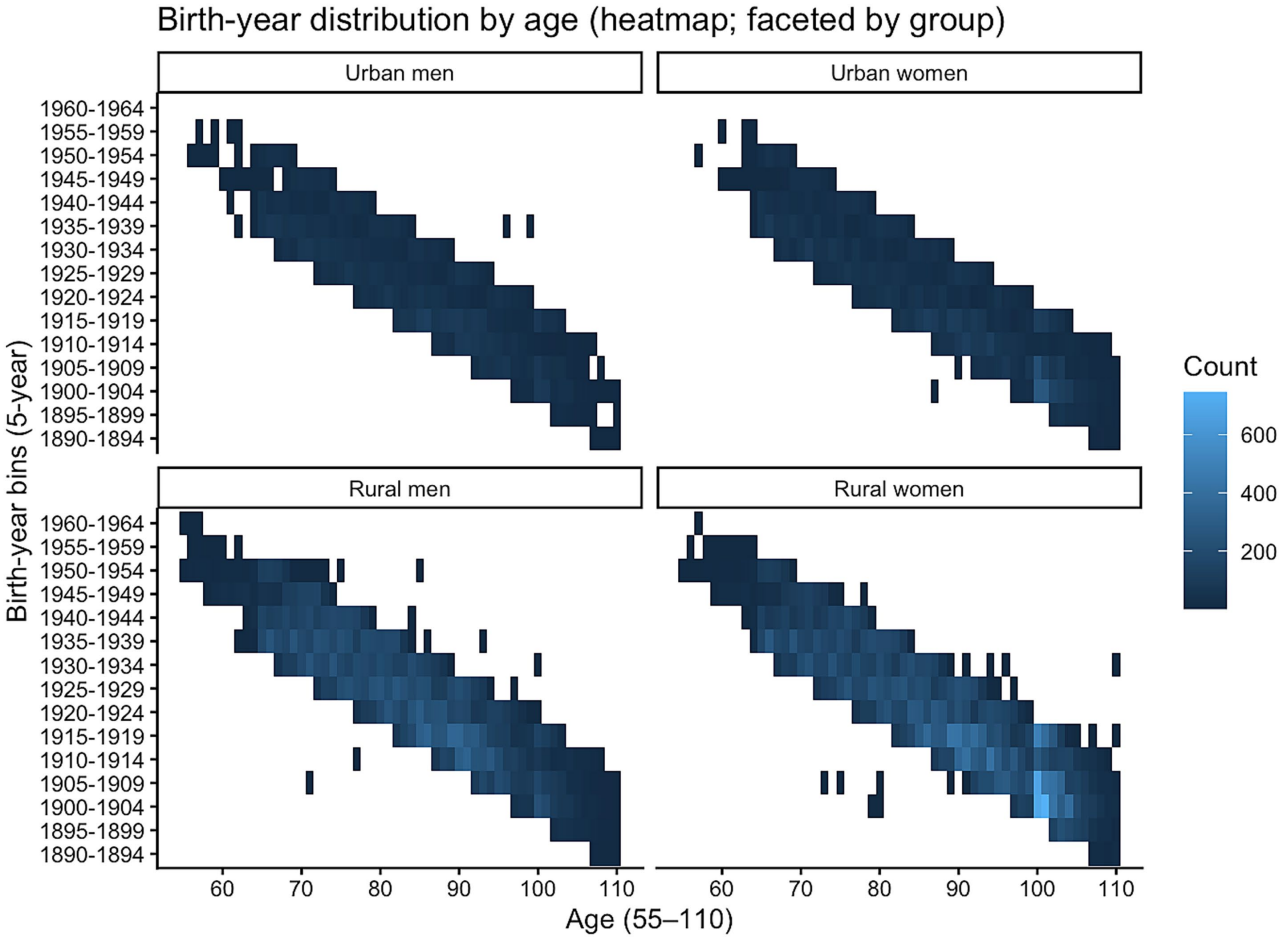
Heat map of age × birth cohort.

To assess the identifiability of the age and cohort effects, we plot heat maps of age and birth cohort by subgroup, as shown in Figure 3. Figure 3 indicates that observations are concentrated along an age–cohort diagonal band, and that the cohort-coverage width at a given age differs across subgroups. A thinner diagonal band (i.e., lower cohort diversity at the same age) is less conducive to jointly estimating age and cohort effects, thereby increasing model uncertainty. Conversely, a thicker diagonal band implies that multiple birth cohorts are observed at the same age; cross-cohort differences in FI provide information for identifying cohort effects and, in turn, support more stable identification of the age effect.

### 4.2 Estimation of the three effects

**Figure 4:**
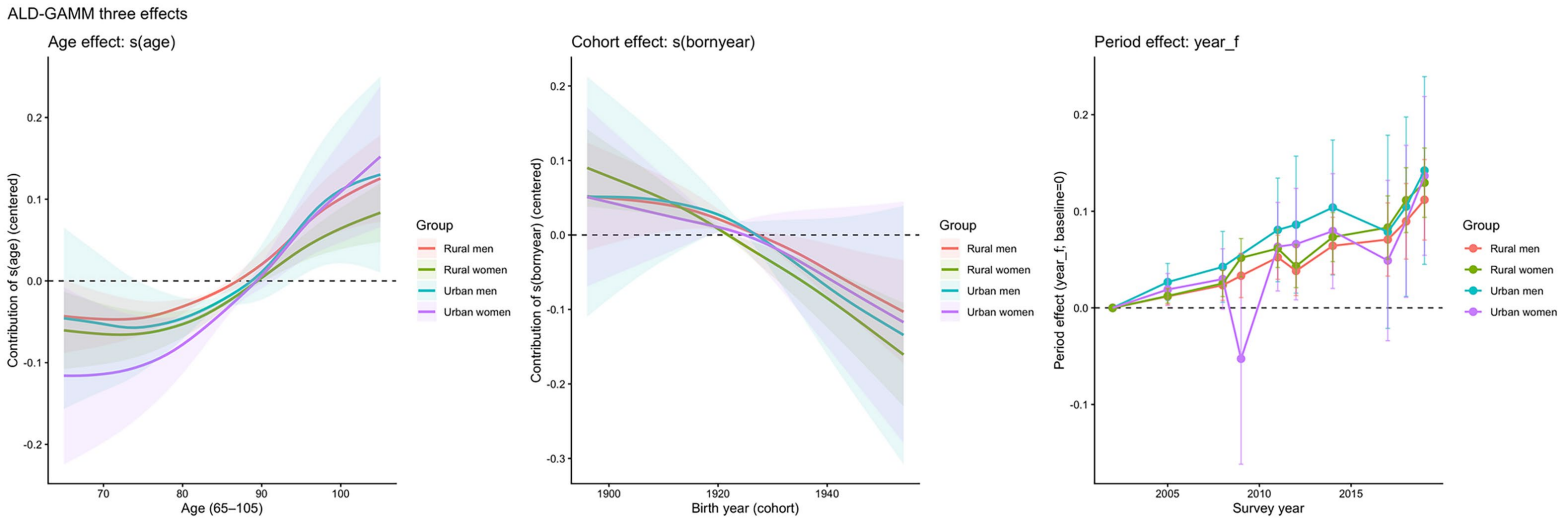
The three effects estimated from the decomposition.

Based on the methods described above, we present the three effects obtained from the model-based decomposition in Figure 4. In Figure 4, the horizontal axis denotes age, and the vertical axis represents the relative contribution of the corresponding effect. The four curves correspond to the four subpopulations: urban men, urban women, rural men, and rural women. The results in Figure 4 reveal the structural sources of frailty variation among older adults in China as it evolves with the life course, cohort succession, and changing historical contexts.

Figure 4A reports the estimated age effect after decomposition. Between ages 65 and roughly 85, the age contribution is negative, indicating that—after controlling for cohort and period effects—the FI for this age range is below the overall population average. As age increases, the curve rises steadily and turns positive around age 90. In advanced old age, the contribution of age per se exceeds the average level, and the rate of increase accelerates with age, reflecting the cumulative accumulation of functional deficits and the cascading rise of multimorbidity associated with ageing. Across subpopulations, urban women exhibit the lowest curve at younger-old ages, with a larger negative deviation, but their curve rises more rapidly at older ages and reaches a higher positive contribution at the upper end of the age range. This suggests that age-related frailty growth is most pronounced among urban women. Rural women show a smaller increase at advanced ages and a flatter overall profile, implying a weaker net age-related increment. The amplitude of change in the age-effect curves for urban and rural men is also larger than that for rural men.

Figure 4B presents the estimated cohort effect. The cohort effect declines as birth year increases, implying that earlier-born cohorts make a positive contribution relative to later-born cohorts. In other words, conditional on the same age and the same survey period, older adults from later-born cohorts exhibit systematically lower frailty levels, consistent with the net effects of cumulative intergenerational improvements in health capital—such as better early-life nutrition, reduced exposure to adverse public health environments, and improved access to medical care. Unlike the age effect, the cohort gradient is steepest for rural women: early-born cohorts show clearly positive contributions, whereas the most recent cohorts display the largest negative contributions, indicating particularly pronounced intergenerational improvement among rural women. The other three groups also show downward trends but with flatter gradients. Moreover, the cohort-effect curves for urban men and urban women largely overlap at the early-cohort end, suggesting that within urban areas, gender differences may be expressed more strongly through age and period dynamics than through cohort replacement.

Figure 4C shows the estimated period effect, where the vertical axis represents fixed-effect deviations relative to the baseline survey year. The period coefficients for all four groups exhibit a shared upward trend. Over the survey period covered by the sample, the overall FI level among older adults shifts upward, reflecting changes in the macro environment and in survival composition.

Table 3 reports model diagnostic and significance testing results. As shown in Table 3, all three APC components of the frailty index obtained from the decomposition are statistically significant.

**Table 3:** Tests of model significance.

Group	s(age)	s(bornyear)	year_f
Urban_M	<0.001(***)	<0.001(***)	<0.001(***)
Urban_F	<0.001(***)	<0.001(***)	<0.001(***)
Rural_M	<0.001(***)	<0.001(***)	<0.001(***)
Rural_F	<0.001(***)	<0.001(***)	<0.001(***)

*** indicates significance at the 0.1% level.

### 4.3 FI–age trajectories and subgroup comparisons

**Figure 5:**
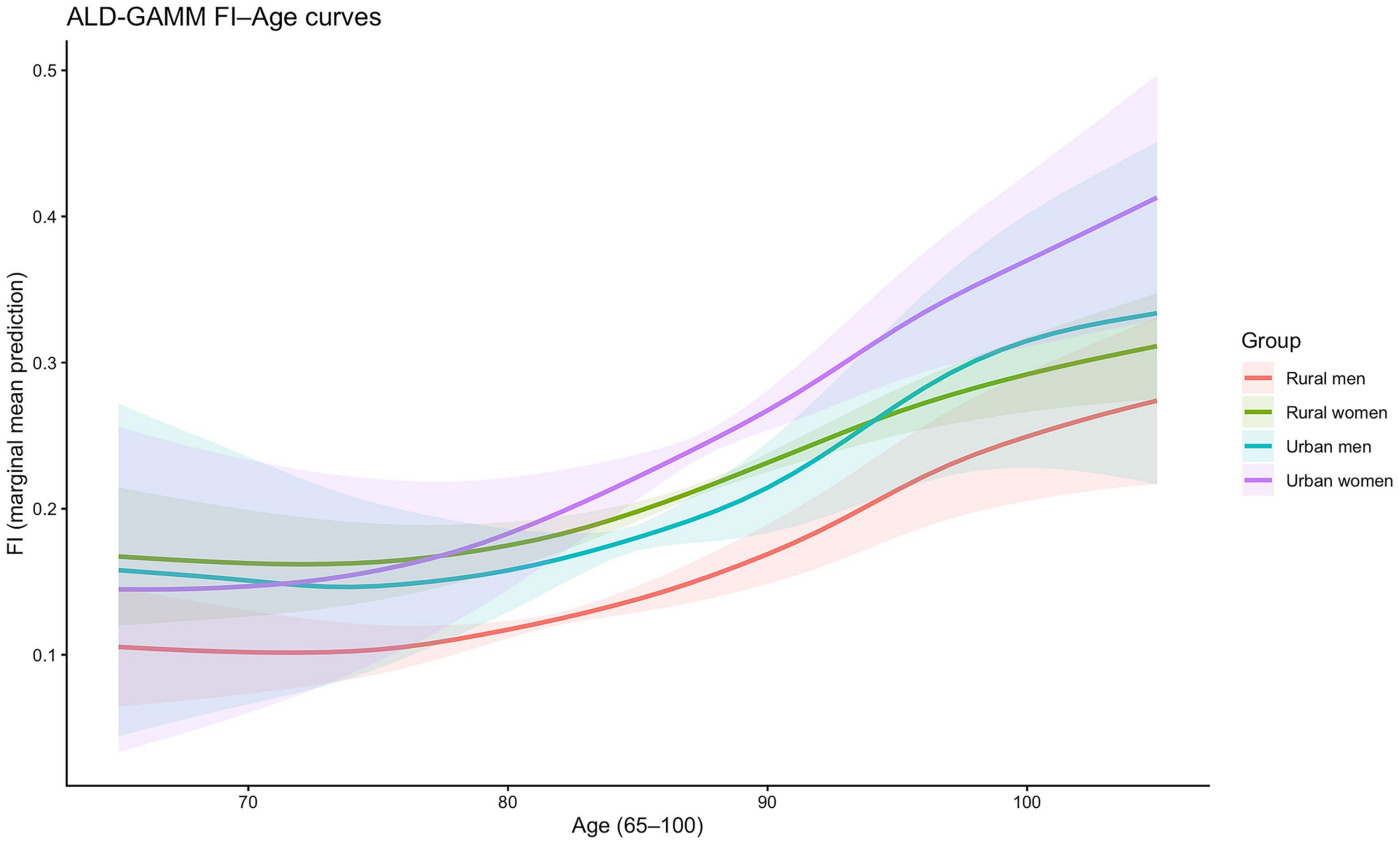
Marginalized FI–age trajectories.

Building on the separate identification of age, cohort, and period effects in the previous section, we obtain the FI–age trajectories for the four subpopulations by marginalizing cohort and period effects using observation-weighted averages. Each curve represents the population-level mean pathway of frailty change with age after accounting for differences across birth cohorts and survey periods within the selected sample. The results are shown in Figure 5.

As indicated by the overall trends in Figure 5, all four groups display stage-specific features in early old age, when health selection, survivor advantage, and the accumulation of frailty have not yet fully manifested. Consequently, the frailty index is relatively flat—or even slightly concave—around ages 65–75. Upon entering the oldest-old stage, the slope increases markedly: deficit accumulation becomes a pervasive process, the pace of accumulation accelerates, and frailty growth becomes steeper, indicating more intense changes in frailty. This pattern suggests that frailty does not increase at a constant linear rate with age and implies systematic divergence in frailty progression speeds across groups at advanced ages.

Between-group comparisons further reveal an age-dependent stratification structure. First, the FI curve for rural men remains the lowest across the entire age range and increases relatively slowly, indicating the lowest average frailty level. In contrast, rural women exhibit higher FI values than rural men at most ages, implying higher frailty among rural women at the same age. Second, urban women show particularly pronounced dynamics: around age 70, the gap between this group and the others is relatively small, but from late middle and early old age onward, the increase in frailty accelerates substantially, and after age 90 urban women maintain the highest FI levels, with the distance to the other three groups continuing to widen.

Overall, frailty among China’s older population increases with age in a clearly nonlinear manner and accelerates at advanced ages. Urban–rural and gender disparities also exhibit a distinct age dependence, expanding substantially in the oldest-old stage. Urban women bear the heaviest frailty burden, whereas rural men consistently exhibit the lowest levels.

**Figure 6:**
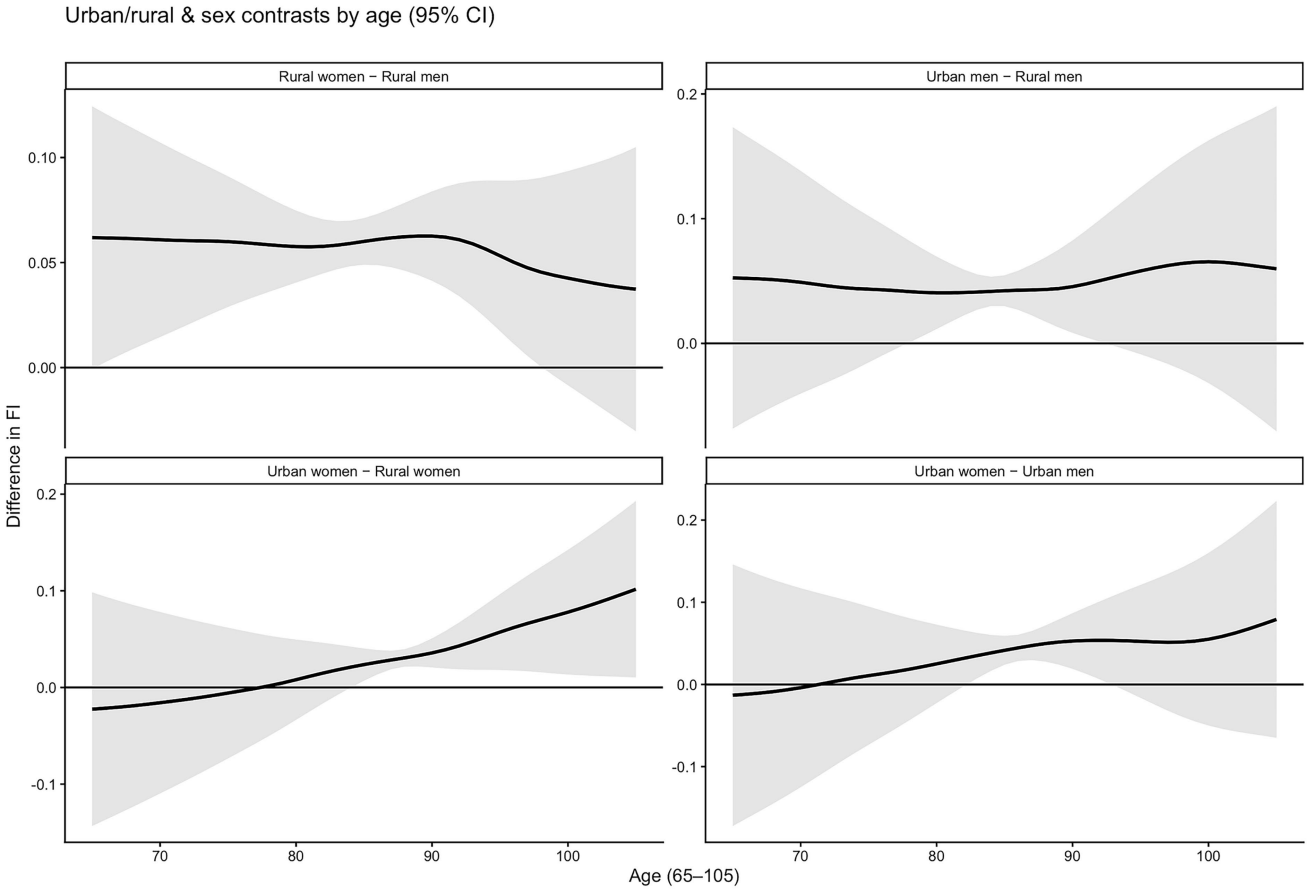
Urban–rural and gender differences by age.

Figure 6 compares frailty differences by urban–rural residence and gender among older adults in China. The vertical axis reports the between-group differences in FI. The four panels present two main-effect contrasts: rural women versus rural men, urban men versus rural men, urban women versus rural women, and urban women versus urban men. Based on the significance tests, the differences shown in these four panels are statistically significant.

Across the four primary contrast curves, between-group disparities exhibit pronounced age heterogeneity. First, the difference between rural women and rural men is positive after age 65 and remains relatively stable, indicating persistently higher marginal frailty among women within rural areas, with a slight decline at very advanced ages. Second, the difference between urban men and rural men is positive overall, suggesting higher frailty among urban men than rural men. This gap gradually increases at advanced ages, implying that urban–rural differences among men are more salient in later life. Third, comparing urban women with rural women shows limited urban–rural disparity at younger ages; however, at advanced ages, frailty rises more rapidly among urban women, leading to an accumulating and widening gap. Finally, the difference between urban women and urban men is positive overall and expands progressively with age, indicating that within urban settings, women exhibit increasingly higher frailty than men at older ages, especially in advanced old age. Taken together, advanced old age constitutes a critical interval in which urban–rural and gender disparities jointly amplify, with this amplification occurring more prominently among women and within urban contexts.

Table 4 reports tests of urban–rural and gender differences. The results indicate statistically significant urban–rural and gender disparities in frailty dynamics among China’s older population.

**Table 4:** Significance tests for urban–rural and gender differences.

Contrast	*P*-value
Urban–Rural (Men)	<0.001 (***)
Urban–Rural (Women)	<0.001 (***)
Women-Men (Urban)	<0.001 (***)
Women-Men (Rural)	<0.001 (***)

*** indicates significance at the 0.1% level.

## 5 Projecting frailty trends among older adults in China

**Figure 7:**
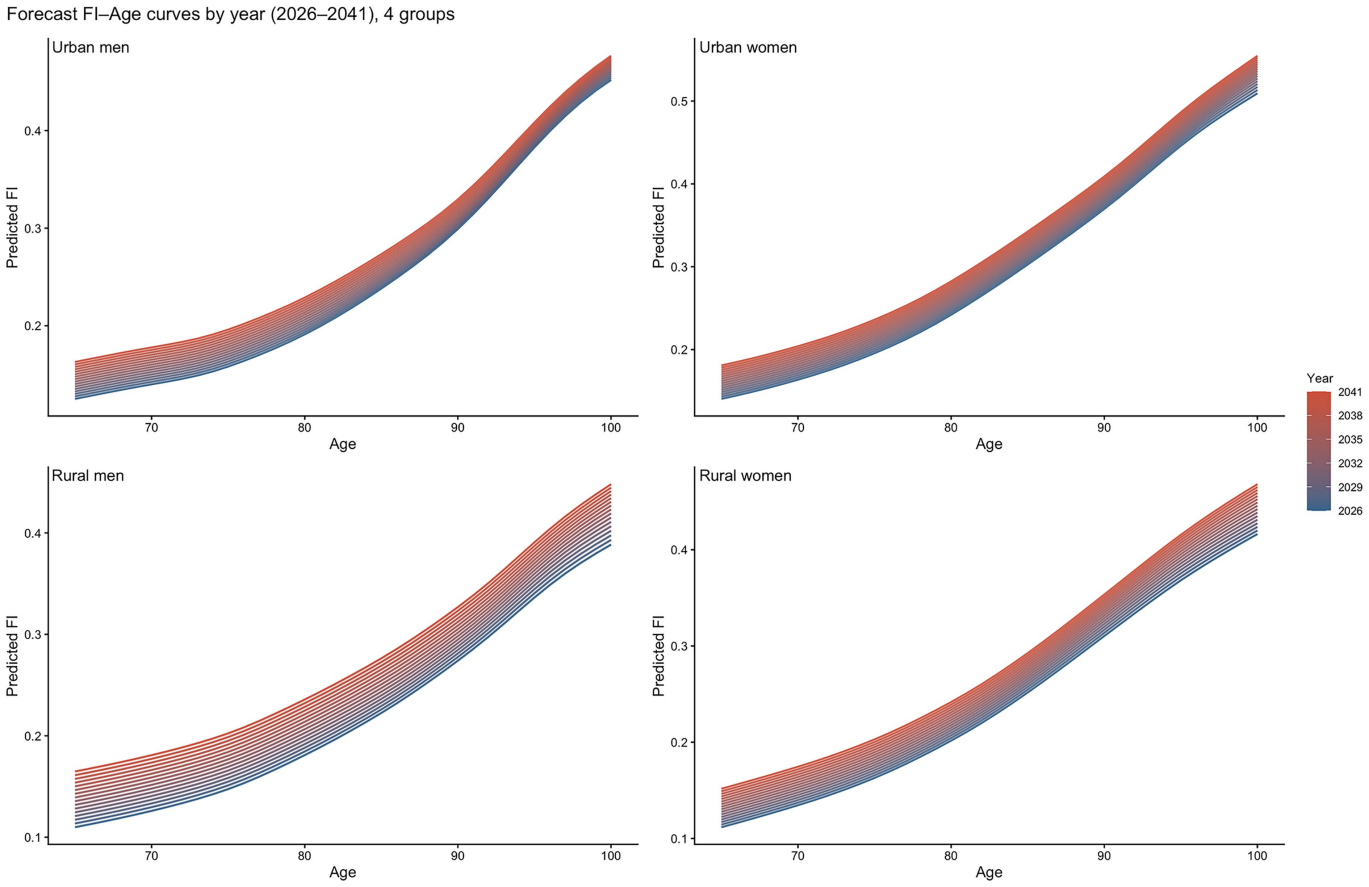
Projected changes in the frailty index by urban–rural residence and gender, 2026–2041.

This section applies the APC decomposition results and analytical framework described above. Future frailty levels are expressed as the superposition of the age trajectory and the extrapolated cohort and period effects. At the population level, we generate projections of both (i) the age distribution of the mean frailty index among China’s future older population and (ii) its overall time trend. The results are presented in Figure 7.

Figure 7 displays projected FI–age curves for the four subpopulations over 2026–2041. The horizontal axis denotes age (given the range of the population projections, we focus on ages 65–100), and different colors correspond to different projection years (with a gradient from earlier to later years). Accordingly, within each panel, the age profile of the curves reflects the accumulation of frailty driven by the age effect, whereas the vertical shifts across years at a fixed age reflect the time drift in overall frailty levels implied by the extrapolated cohort and period effects.

The projections indicate that, from 2026 to 2041, the frailty index for all four groups increases with age and shifts upward over time, suggesting a persistent rise in frailty among older adults during the projection horizon. In subgroup comparisons, the urban–rural disparity emerges as a salient structural feature: the upward shifts over time are generally larger for rural men and rural women than for their urban counterparts. This pattern is particularly pronounced at advanced ages, implying that frailty among rural older adults not only increases continuously over calendar time but also tends to accelerate in the oldest-old stage. By contrast, although urban men and urban women also exhibit robust upward trends, the year-to-year upward shifts are relatively more modest and the overall trajectories are comparatively smoother.

Overall, the projected growth in the frailty burden is more likely to be concentrated among rural older adults, with stronger cumulative effects and a tendency toward widening disparities at advanced ages.

**Figure 8:**
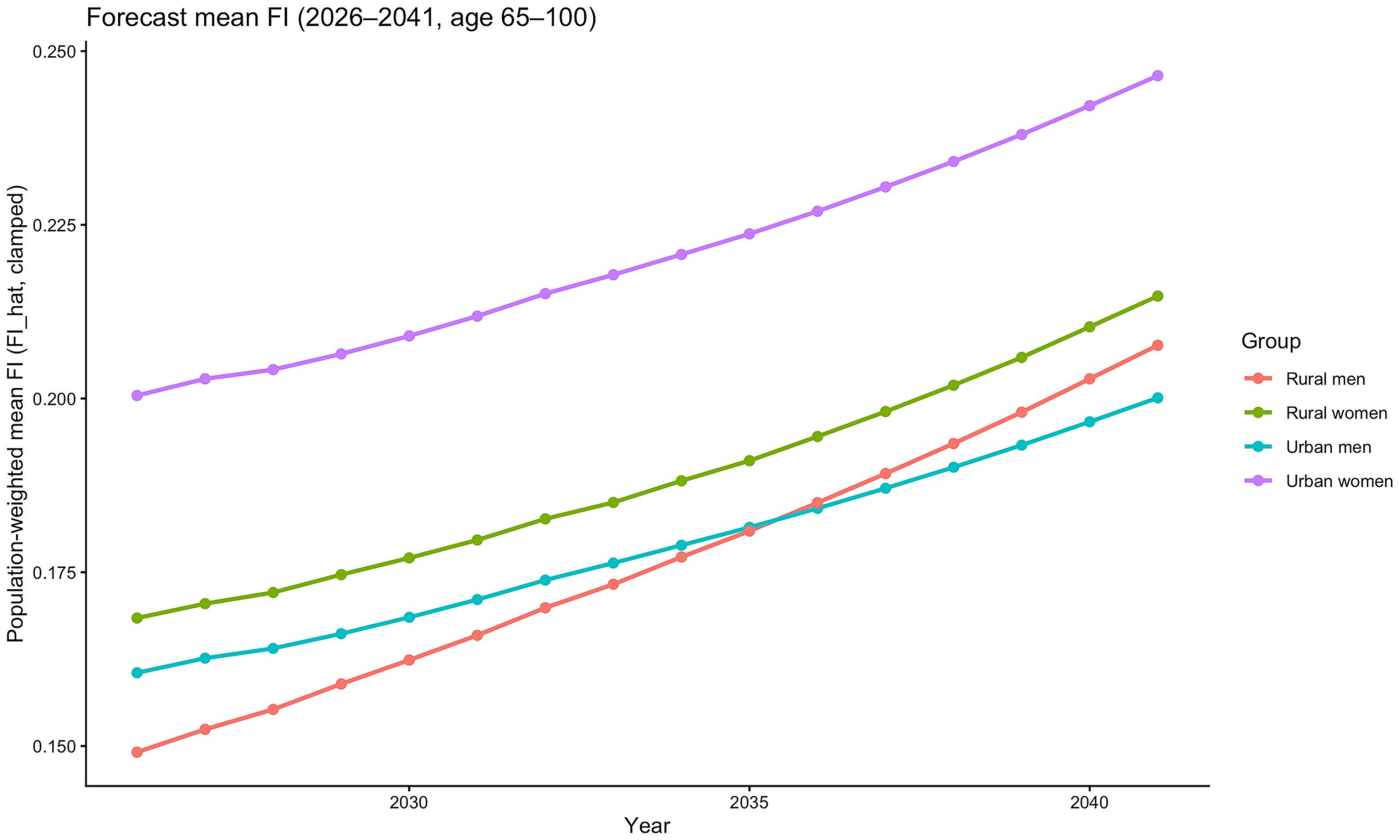
Projected mean frailty index weighted by population projections.

As population ageing in China continues to deepen, the share of older adults at advanced ages is projected to rise further. To quantify the overall frailty burden under deep ageing, we weight the APC-based frailty projections by the projected age structure of the older population and obtain the future trend in the mean frailty index for China’s older adults, as shown in Figure 8.

Figure 8 presents the population-weighted mean FI across the projected age range (the older-population counts are based on projections produced within our research team using the cohort-component method). It can therefore be interpreted as the overall frailty burden—and its evolution over time—for each urban–rural and gender subgroup during the projection period. The results indicate that, from 2026 to 2041, the mean FI increases monotonically for all four groups. Within the given age-structure range, the frailty burden continues to accumulate over time, but the slopes differ markedly across groups, revealing structural inequality in both levels and growth rates. Urban women remain at the highest level throughout the projection horizon, following a relatively steady upward trajectory, implying a persistently high overall frailty burden. Rural women form the second tier, with levels substantially higher than those of both male groups and with a more pronounced increase. Thus, women are projected to face a dual pressure of higher frailty levels and sustained deterioration over time. The two male groups exhibit distinct dynamics: at the beginning of the projection period, urban men have a higher mean FI than rural men; however, rural men show a steeper upward slope, and by the middle of the projection horizon the mean FI among rural men surpasses that of urban men.

Overall, the trends in Figure 8 clearly indicate that the projected rise in frailty burden is not evenly distributed: women have higher levels than men, and rural groups—especially rural men—experience faster growth.

## 6 Discussion and conclusion

Situated in the real-world context of China’s deeply ageing society, this study focuses on the structural evolution and future trajectory of frailty among older adults. From a demographic perspective, and drawing on the logic of accelerated longitudinal design (ALD), we move beyond treating frailty as a purely descriptive indicator of individual health status. Instead, we conceptualize it as a decomposable, comparable, and extrapolatable object that can reveal three mechanisms—age accumulation, cohort succession, and macro-environmental shocks. We provide differentiated evidence and trend assessments for four subpopulations defined by urban–rural residence and gender.

First, along the age dimension, age is a key determinant of frailty. Frailty increases relatively slowly in early old age, but the slope rises markedly at advanced ages, where deficit accumulation accelerates. Along the cohort dimension, the birth-cohort effect shows an overall downward trend. Later-born cohorts exhibit systematically lower frailty at the same age and in the same period, indicating that cumulative intergenerational improvements in health capital continue to yield an observable net effect in later life. Along the period dimension, the period effect shifts upward over the sample window, suggesting that the overall frailty level among older adults continues to rise across survey years. This implies that macro-level environmental and institutional changes may exert complex influences on composite health status: their direction is not necessarily aligned with a simple, one-way health gain from improved medical conditions, and may instead be linked to mechanisms such as changes in survival composition, extended survival with chronic disease, and the expansion of unhealthy life expectancy.

Second, based on observation-weighted marginalization over cohort and period heterogeneity, frailty displays pronounced urban–rural and gender disparities. Across later life, the four subpopulations form a relatively stable ranking: rural men remain at the lowest level overall, whereas urban women increase more rapidly at advanced ages and ultimately maintain the highest frailty levels. Moreover, the urban–rural gap among women widens with age faster than the corresponding gap among men, and the strengthening of gender differences within urban contexts proceeds faster than within rural contexts. Consequently, advanced old age becomes a critical interval in which urban–rural inequality and gender inequality interact and amplify.

Third, projections for 2026–2041 indicate that frailty will continue to increase in all four subpopulations, with substantial divergence in growth rates. The year-to-year upward shifts for rural groups (rural men and rural women) are generally larger than those for urban groups, and this difference is more pronounced at advanced ages, implying that future frailty pressures may be released more intensively among rural older adults. Population-weighted mean results further show that urban women remain at the highest level throughout the projection horizon and continue to increase steadily. Although urban men start above rural men, rural men exhibit a steeper slope and surpass urban men after the mid-point of the projection period, forming a dynamic pattern of faster growth among rural men.

Several limitations should be noted. The analysis is based on the surviving population observed at survey waves rather than complete birth-cohort populations. Given the strong association between frailty and mortality risk, results at advanced ages are inevitably affected by selective survival; moreover, differences in mortality selection across social groups may yield more complex patterns in between-group comparisons at the oldest ages. In addition, under limited information, our projections rely on tail-linear extrapolation for cohort effects and smooth extrapolation for period effects. This strategy helps reduce the risk of extreme extrapolation, but it remains a scenario exercise that extends existing structural trends. Future research could incorporate mechanism variables—such as access to health services, social security coverage, lifestyles, and community environments—to conduct structural scenario comparisons.

Based on these findings, we argue that health governance in a deeply ageing society should further shift from disease-centered single-condition management toward integrated governance centered on functional decline and care-related risks, using frailty as a bridging indicator linking public health, medical services, and long-term care systems. The faster projected growth among rural older adults implies that resource allocation should not rely solely on static layouts based on current level differences; rather, it should emphasize forward-looking responses to changing frailty pressures. Meanwhile, the persistently high and rising trajectory among urban women suggests that gender disparities are not simply a matter of “higher levels among women,” but may reflect the concentration of chronic deficit accumulation and care needs under longer survival. This calls for more targeted institutional responses in areas such as community support, rehabilitation services, and the reduction of family caregiving burdens.

## Data Availability

The original contributions presented in the study are included in the article/Supplementary material, further inquiries can be directed to the corresponding author.
