# Supplementary material for: Age-period-cohort decomposition and projections of the frailty index among older adults in China
Source: Front Public Health. 2026 Apr 15;14:1806784. doi: 10.3389/fpubh.2026.1806784 (PMC13124577; doi:10.3389/fpubh.2026.1806784)
Supplement: Supplementary file 1 [file Table_1.DOCX]

Supplementary Table 1. Sensitivity Analysis Results for Smoothing Parameters

| group_label | age_max_abs_diff_max | age_corr_min | cohort_max_abs_diff_max | cohort_corr_min | period_max_abs_diff_max | period_corr_min |
| --- | --- | --- | --- | --- | --- | --- |
| Rural women | 0.0001 | 1 | 0.002 | 1 | 0 | 1 |
| Rural men | 0.0004 | 1 | 0.0003 | 1 | 0 | 1 |
| Urban women | 0.0002 | 1 | 0.0002 | 1 | 0.0001 | 1 |
| Urban men | 0.0005 | 1 | 0.0002 | 1 | 0.0001 | 1 |
